# Exploring the molecular basis of age-related disease comorbidities using a multi-omics graphical model

**DOI:** 10.1038/srep37646

**Published:** 2016-11-25

**Authors:** Jonas Zierer, Tess Pallister, Pei-Chien Tsai, Jan Krumsiek, Jordana T. Bell, Gordan Lauc, Tim D Spector, Cristina Menni, Gabi Kastenmüller

**Affiliations:** 1 Department of Twin Research and Genetic Epidemiology, Kings College London, London, United Kingdom; 2 Institute of Bioinformatics and Systems Biology, Helmholtz Zentrum München, Neuherberg, Germany; 3 Institute of Computational Biology, Helmholtz Zentrum München, Neuherberg, Germany; 4 German Center for Diabetes Research (DZD), Neuherberg, Germany; 5 Genos Glycoscience Research Laboratory, Zagreb, Croatia

## Abstract

Although association studies have unveiled numerous correlations of biochemical markers with age and age-related diseases, we still lack an understanding of their mutual dependencies. To find molecular pathways that underlie age-related diseases as well as their comorbidities, we integrated aging markers from four different high-throughput omics datasets, namely epigenomics, transcriptomics, glycomics and metabolomics, with a comprehensive set of disease phenotypes from 510 participants of the TwinsUK cohort. We used graphical random forests to assess conditional dependencies between omics markers and phenotypes while eliminating mediated associations. Applying this novel approach for multi-omics data integration yields a model consisting of seven modules that represent distinct aspects of aging. These modules are connected by hubs that potentially trigger comorbidities of age-related diseases. As an example, we identified urate as one of these key players mediating the comorbidity of renal disease with body composition and obesity. Body composition variables are in turn associated with inflammatory IgG markers, mediated by the expression of the hormone oxytocin. Thus, oxytocin potentially contributes to the development of chronic low-grade inflammation, which often accompanies obesity. Our multi-omics graphical model demonstrates the interconnectivity of age-related diseases and highlights molecular markers of the aging process that might drive disease comorbidities.

Aging is a multi-factorial process that affects the entire organism, thus causing decreased fitness, disease and eventually death. As the population of western countries is aging^1^, the prevalence of a variety of age-related diseases, such as cardiovascular disease, cancer^2^ and chronic kidney disease (CKD)^3^ and many related diseases are increasing. Finding mechanisms that cause diseases with progressing age as well as better understanding disease comorbidity patterns is thus essential to counteract an explosion of health care costs. Epidemiological studies have already identified a broad spectrum of molecules associated with aging from various layers of biology accessible through modern omics technologies^4^. These molecules include epigenetic markers^5^, RNA abundances^6^, protein abundances^7^, post-translational protein modifications – such as protein glycosylation^8^ – and metabolite concentrations^9^. However, these studies analyzed omics datasets independently, thus neglecting the intrinsic interactions of biological entities within and across omics layers. Taking into account this complex interplay is necessary to unveil the causal structure of multi-factorial processes such as aging.

Various concepts have been proposed to integrate data from different molecular layers and (omics) technologies in systems biology and the newly emerging fields of systems genetics^10^ and systems medicine^11^. Thereby, networks have been shown to be particularly useful to assess complex interactions in a dataset and to illustrate multivariate dependencies^12^. As an example, using network approaches, it was demonstrated that co-occurring diseases are linked to mutations in the same gene^13^, in genes that interact with each other^14^, or in genes that are involved in the same metabolic pathway^15^, explaining observed patterns of comorbidity^16^.

Due to the increasing availability of high-dimensional omics datasets, networks can now be inferred also directly from measured data facilitating the unbiased analysis of specific conditions of interest, independently of prior knowledge. For instance, gene co-expression networks were used to analyze the influence of anti-cancer drugs on gene expression^17^. Integration of such co-expression networks with other omics layers allowed for prioritization of interesting, potentially causal, targets^18^. Even though these types of correlation-based networks led to a wide range of discoveries, they suffer from vast numbers of spurious correlations that inflate the number of edges and obscure the underlying mechanisms. Conditional independence graphs, such as graphical models^19^, were proposed as solution to overcome the problem of mediated associations^20^ by revealing the relevant direct associations between variables. Although the direction of the associations and, thus, causality cannot be determined by these models in most cases, the resulting network of direct associations between variables can be considered as the undirected skeleton of their underlying causal structure. While the proposed graphical models are well established for multi-variate Gaussian distributed data, the extension to mixed distributions, as commonly observed in phenomics data (e.g. gender, disease states), is substantially more complex^21^.

In this study, we aimed to investigate the molecular basis of age-related diseases and its influence on disease comorbidities. To this end, we used an integrated mixed graphical model (MGM) approach to combine aging markers from four different high-throughput omics datasets on the same individuals, namely epigenomics, transcriptomics, glycomics and metabolomics, together with extensive phenotypic data. While we cannot infer actual causality using MGM, the resulting network of direct associations that are independent of all other variables within the model is expected to provide valuable insights into the direct molecular interdependencies between various age-related phenotypes. To the best of our knowledge, this is the first study that uses graphical models to combine data from multiple molecular omics and phenomics datasets.

## Results

**Figure 1:**
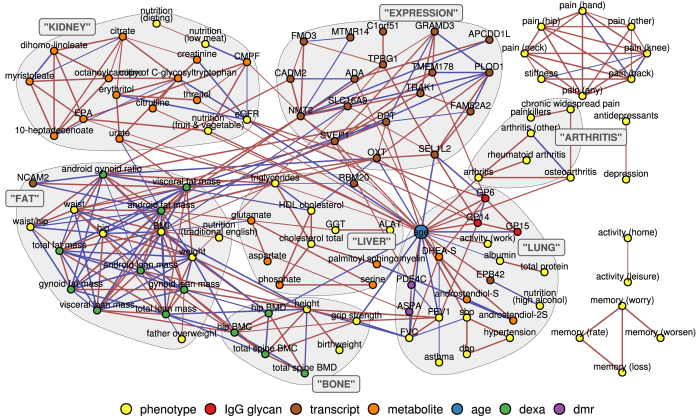
Multi-Omics MGM of Age. Each node in the graph represents one age-related variable. Omics markers were selected according to literature from epigenomics (purple), transcriptomics (brown), glycomics (red) and metabolomics (orange) datasets and combined with DXA measurements (green) and other clinical phenotypes (yellow). Edges between nodes were inferred using a mixed graphical model approach, and thus indicate the conditional dependence between variables; the color represents positive (red) and negative (blue) correlation. An unbiased cluster detection algorithm was used to identify densely connected modules within the network, indicated by grey borders.

We inferred a mixed graphical model using observational data from a cohort of 510 women, aged between 34 and 84, integrating selected age-associated markers from four different omics datasets (see Materials and Methods) with 92 clinically assessed phenotypes (Supplementary Fig. S1). The final model consists of 145 nodes and 316 undirected edges connecting them (Fig. 1). Thus, it is much sparser (316 edges instead of 1900) than a regular correlation graph based on significant pairwise correlations of variables from the same dataset (Supplementary Fig. S2). Most of the nodes (96) form one large connected component, which we refer to as *age-mgm* in the following. There are two smaller components of 8 and 4 nodes that contain variables related to pain and memory function, respectively, two isolated pairs of nodes and 33 unconnected nodes. The degree, betweenness and clustering coefficients of all nodes in the network are presented in Supplementary Tables S1 and S2.

### Topological Properties of age-mgm

The large connected component age-mgm contains 96 variables including age, along with variables from all four omics datasets, and 286 edges connecting them. It has an average node degree of 6.0, an average local clustering coefficient of 46.6% and an average shortest path length of 3.2. Also, its small world index, as defined by Humphries and Gurney^22^, is 6.1 and so the age-mgm can be considered a small-world network with high local clustering and short path lengths. Removing age from the network does not reduce the small worldness of the network. In comparison, the correlation graph, restricted to the same vertices as in the age-mgm, has a just slightly higher clustering coefficient of 57.0% despite the much higher average node degree of 31.2, which results in a small world-index of only 1.7.

**Figure 2:**
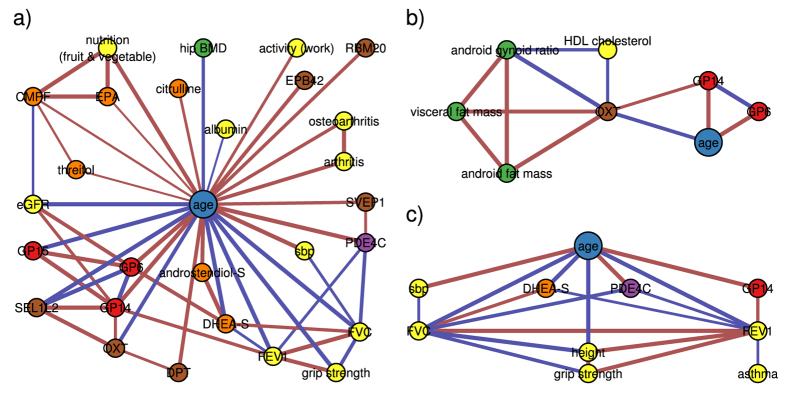
Selected modules from the graphical model. Each panel shows one subgraph from the age-mgm (Fig. 1). (**a**) The direct neighborhood of chronological age. (**b**) The hormone oxytocin (OXT) mediates association of fat mass variables with age as well as the IgG glycosylation marker GP14. (**c**) The direct neighborhood of the lung function measures *forced expiratory volume in one second* (FEV1) and *forced vital capacity* (FVC) contains three omics markers: dehydroepiandrosterone-sulfate (DHEA-S), phosphodiesterase 4 C (PDE4C) and the glycan peak 14 (GP14).

As expected, age is the most densely connected node with a degree of 27 (Fig. 2a). It has a low clustering coefficient (8.0%) but high betweenness (47.5%) centrality. This indicates that age connects different clusters, while its neighbors tend to be unconnected. With an average shortest path length of 2.1 age is also the most central node in the age-mgm.

### Modularity of the age-mgm

There are more edges between variables originating from the same omics dataset than edges connecting them. Particularly transcriptomics and metabolomics variables form dense clusters with 37 and 34 edges within them, respectively. In contrast, only 7 edges connect transcriptomics and metabolomics variables with variables from other omics sets. Similarly, the body composition variables measured by *dual-energy X-ray absorptiometry* (DXA) are densely connected with 45 edges between them (Supplementary Fig. S3).

In order to analyze the graph structure in an unbiased way we used a modularity-based algorithm for cluster detection. This approach yielded seven modules (Fig. 1). The first cluster (EXPRESSION) contains all but three gene expression markers. It is connected with neighboring clusters mainly via expression levels of OXT, which has 6 edges outside of its cluster (Fig. 2b), and SVEP1, which has the highest betweenness centrality (10.5%) within the cluster. The second cluster (LUNG) contains age and several of its direct neighbors from different omics layers. The lung function parameters *forced expiratory volume in one second* (FEV1) and f*orced vital capacity* (FVC) are the most densely connected phenotypes in the cluster (degree 8 and 7 respectively). Both are embedded in a tight cluster with local clustering coefficients of 35.7% and 47.6%, respectively (Fig. 2c). Age is also connected to another small cluster of arthritis-related variables (ARTHRITIS). The body composition variables fall in two different clusters, one of them containing bone density-related variables (BONE) and the other fat and lean mass-related variables (FAT). While the BONE cluster is densely connected with the LUNG cluster, all connections between the FAT cluster and the LUNG cluster, containing the age variable, are mediated, mainly via gene expression variables from adipose tissue. The next cluster (LIVER) contains the liver markers *alanine-aminotransferase* (ALAT) and *gamma-glutamyl transpeptidase* (GGT) along with cholesterol and triglyceride levels and several amino acids. It also contains the gene expression marker of the RBM20 gene that mediates the connection of the cluster with age and the LUNG cluster. The last cluster (KIDNEY) contains mainly metabolite levels, but also markers for nutrition and a measure of renal function, the *estimated glomerular filtration rate* (eGFR). With 9 edges, C-glycosyltryptophan is central within the metabolite cluster. However, the eGFR (degree 7) is the main connection of the metabolomics cluster with age as well as IgG glycosylation markers. The only connections of the renal cluster with other clusters, apart from the LUNG cluster via age, are edges between urate and the FAT and LIVER clusters.

### Robustness of the age-mgm

For estimating the robustness of our model, we inferred additional networks based on different cutoffs for edge inclusion and on changed selection of omics variables. Comparing these networks with our original model, we found node centrality as well as module assignments to be stable when varying the cutoff for edge inclusion (Supplementary Fig. S4). The modules identified in the original age-mgm remained stable even when including all available metabolomics variables with known chemical identity, i.e. when basing the inference procedure on 196 metabolites in addition to the 23 pre-selected ones (Supplementary Table S4).

To investigate the reproducibility of the inferred age-mgm for a different set of samples we determined two separate models from disjoint datasets incorporating the first and second twin of each family, respectively, and compared these models to the original network. The two resulting models reproduce 93.5% of all edges, with only 21 edges being unique to the initial model (Supplementary Fig. S5A, Supplementary Dataset S1). Moreover, in the models of the two disjoint datasets these unique edges just missed the edge inclusion cutoff of 80% in most cases, which is most likely due to the reduced power in the smaller datasets (Supplementary Fig. S5B).

## Discussion

In this study, we inferred a robust graphical multi-omics model of age-related diseases by integrating disease phenotypes with molecular markers from four omics layers based on data available for 510 women from the TwinsUK cohort. Despite the sparsity of our model, which omits mediated associations, most variables form one connected component (age-mgm) consisting of seven modules. Interestingly, each of these modules represents a different aspect of aging, such as metabolic aging linked to decline of renal function (KIDNEY cluster). Other aspects of aging include the change in body composition, which can be divided in the change of fat and lean tissue (FAT cluster), along with the closely related changes of gene expression in adipose tissue (EXPRESSION cluster), on the one hand, and the decrease of *bone mineral density* (BMD) and *bone mineral content* (BMC) on the other hand (BONE cluster) (Fig. 1).

**Figure 3:**
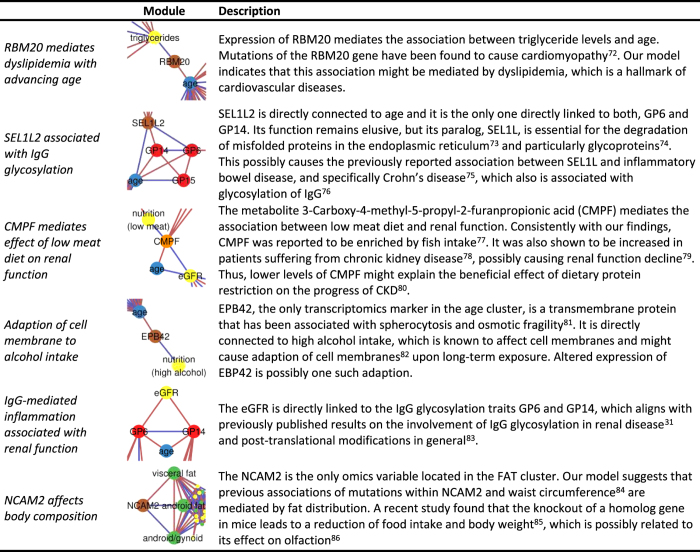
Additional implications of the age-mgm. The figure summarizes selected conclusions drawn from our age-mgm that are not discussed in detail in the main text. Each panel shows a small excerpt from the network, restricted to relevant nodes and edges. Coloring is consistent with Fig. 1.

Our model illustrates multivariate dependencies of age-related diseases that potentially explain comorbidity patterns. Edges in our model represent conditional dependence between two variables, while the absence of an edge implies their conditional independence given all other variables in the model. Specifically, this means that previously observed age-associations of the variables, which are not directly linked to age in our model, occur due to the mediation by other variables between them. This differentiation between mediated and direct associations allows us to draw conclusions on underlying mechanisms even though the causal directions cannot be inferred. In the following section we will discuss some key findings from our aging model in detail. Figure 3 summarizes additional hypotheses derived from the model, which we will not further discuss for the sake of brevity.

### Lung Function is a Central Aging Process

Lung function appears to be a central aging phenotype in our age-mgm. Both lung function measures, FEV1 and FVC, are directly connected with age and are, besides age, the most densely connected nodes in the LUNG cluster, connected with three different omics markers (Fig. 2c): (i) The metabolite dehydroepiandrosterone sulfate (DHEA-S) is one of the most abundant hormones in humans and well known to decrease with age^23^ and was even suggested as an anti-aging drug^24^. Moreover, DHEA-S has been found to prevent and even revert pulmonary hypertension in rats^25^, suggesting a causal effect of DHEA-S on lung function. (ii) The methylation probe cg17861230 lies in the PDE4C gene, an enzyme that catalyzes the hydrolysation of cAMP. Expression levels of PDE4C were previously found to be associated with lung function^26^. PDE4 is a potential target for drugs against COPD and a PDE4 inhibitor, Roflumilast, has been approved by the EMA for treatment of COPD^27^. In this example, our graphical model approach indeed unveiled a known causal interaction of variables while removing less relevant mediated associations. (iii) Finally, the IgG glycosylation marker GP14 is connected to lung function in the age-mgm. GP14 is a glycan structure with terminal galactose, which is known to change the inflammatory state of IgG^28^. While defects of general protein glycosylation^29^ as well as an involvement of IgG^30^ in COPD have been previously reported, glycosylation of IgG has so far not been associated with lung function. Our model suggests a contribution of IgG mediated inflammation and might help to unveil mechanisms of lung disease in dedicated experiments. As IgG glycosylation is also related with kidney function in our age-mgm as well as in previous studies^31^, this might provide an explanation for the comorbidity of lung disease and renal disease.

### Decline of Renal Function Links Age with Metabolic Shift

The blood metabolome was shown to be strongly influenced by age in several studies^9^. In the age-mgm most of the age-associated metabolites (13) form one large cluster with only four of them being directly linked to age, while the remaining nine metabolites are only indirectly associated with age. For six of these nine metabolites the shortest path to age is through eGFR, a measure of renal function. Even though our model is undirected, age is the only non-modifiable variable in our model. We thus hypothesize that with increasing age renal function declines leading to the major shift in the aging blood metabolome, which possibly causes further diseases.

### Urate Mediates Association of Renal Function with Body Composition

Urate mediates the connection of the KIDNEY cluster with FAT and LIVER clusters. Hyperuricemia has been previously reported to be associated with obesity, particularly increased visceral fat mass^32^, and increased triglyceride levels^33^, which appears to be a direct association according to our model. Indeed, there is evidence that urate actually contributes to the development of obesity and diabetes, rather than being just a consequence of obesity: Elevated serum levels of urate were found to predict, amongst others, obesity^34^ and diabetes^35^. By knocking out the uric acid transporter SLC2A9 in mice, DeBosch and colleagues found that hyperuricemia causes several phenotypes of the metabolic syndrome, including obesity, dyslipidemia and hypertension^36^. Administering a compensating treatment attenuated some but not all of the observed symptoms. Hyperuricemia is also a known comorbidity of renal disease, however the causal direction of this association is controversial^37^. Renal disease and uremia were also shown to affect the gut microbiome composition^38^, which is known to be strongly associated with obesity and other symptoms of the metabolic syndrome^39^. Thus, the microbiome is possibly a hidden mediating factor, not included in our model, of the association between hyperuricemia and obesity. Even though its mode of action remains elusive, urate appears to be a key factor for the comorbidity of renal disease and obesity.

### Hormone Expression Directly Associates with Body Composition

It is commonly known that BMI as well as waist and hip circumferences and body fat mass change with age. Nonetheless, we found neither of them directly linked to age in our model. Instead, all associations between age and the fat cluster are mediated. One of the paths connecting the FAT cluster with age is channeled via urate and renal function (as discussed above). A second path leads via the EXPRESSION cluster and, particularly, the expression of oxytocin (OXT) (Fig. 2b), which accordingly mediates 6.0% of all shortest paths in the model. OXT is also directly linked to HDL cholesterol levels. While adipose tissue was traditionally considered as storage tissue, it receives increasing attention as endocrine organ^40^ that amongst others produces OXT. OXT is a hormone with a broad spectrum of functions, ranging from reproductive functions and control of social behavior^41^ to energy metabolism^42^. One common explanation for the influence of OXT on obesity is its effect on food intake^43^, but there is also a diet-independent effect of OXT on the lipid metabolism in adipose tissue^44^. Thus, OXT was suggested as drug against obesity and type 2 diabetes development and has been successfully tested in a first pilot trial^45^. Our results indicate that the age-related change of body composition can, amongst others, be attributed to alterations of gene expression in adipose tissue and particularly to a change in OXT expression, independently of food intake. OXT might also drive common comorbidities of obesity by causing dyslipidemia, which in turn increases the risk of – amongst others – cardiovascular diseases^46^.

### IgG Glycosylation as New Mechanism of Obesity-Associated Inflammation

Obesity is known to be associated with chronic low-grade inflammation and activation of immune function^47^, which is thought to be an important mediator between obesity and common comorbidities, such as type 2 diabetes^48^. In our model the expression of OXT mediates the association of android and visceral fat mass with inflammatory IgG glycosylation. The influence of oxytocin on IgG might be mediated by IL6, which was found to be less expressed due to OXT *in vitro*^49^ and thus causes decreased IgG production in B-cells^50^. Our study confirms an effect of increased fat mass on IgG, mediated by OXT, *in vivo*. Moreover, it provides evidence that OXT also affects IgG glycosylation in addition to its expression, thus altering its inflammatory potential. We hypothesize that this is a new mechanism of obesity-induced inflammation, which appears to be independent from previously identified pathways that are mediated by leptin or adiponectin^51^. Both of them are co-expressed with OXT in our data (Pearson correlation r = 0.2, p = 8.1*10^−9^ and r = −0.29, p = 6.1*10^−17^ respectively), but not associated with any of the IgG glycosylation markers.

We also found IgG-mediated inflammation being directly linked with renal function (Fig. 3), suggesting altered inflammatory potential of IgG as possible mechanism causing comorbidities of renal disease, obesity and related phenotypes of the metabolic syndrome. This supports the theory of “inflammaging”, which proposes chronic low-grade inflammation as mechanism that drives disease onset during aging^52^.

### Limitations and Future Directions

Due to the limited availability of large multi-omics datasets and comprehensive collections of clinical phenotypes, our study is restricted by the relatively small sample size of 510 individuals and, more importantly, we were not able to get access to comparable data from an independent cohort to replicate our results. Also, all of our participants are female. As a consequence, our model and the conclusions drawn from it might be only partly transferable to the entire population. However, more and larger multi-omics dataset will be available in near future, for instance from the UK Biobank or the US Precision Medicine Initiative, which will facilitate subsequent studies using our multi-omics integration approach. For the time being, we could only demonstrate the stability of our results by inferring separate models from two disjoint sets of our own dataset that include only one twin of each twin pair, respectively (Supplementary Fig. S5). The limited number of samples also made prior selection of variables indispensable. This selection can be expected to influences the topology and modularity of the final network model. However, in our study, doubling the number of omics variables by not pre-selecting metabolites from the metabolomics data did result in very similar topology and module assignments in the model (Supplementary Table S4). Also, upcoming larger datasets will allow to overcome this limitation by reducing the dimensionality of the data without relying on variable selection based on prior association analyses. While stability selection controls the family-wise error rate (FWER) of edges in the step of network inference, stability selection cannot quantify the total uncertainty in the model and its downstream analyses. However, analyzing the sensitivity of our approach against variations in the inferred network model (e.g. through different threshold for the selection of edges) demonstrated the stability of our results (Supplementary Fig S4, S5 and Table S4). Finally, our approach allows to detect mediation by variables included in the model and thereby enables differentiation between direct and indirect effects, it does, however, not allow to infer causality. Thus, based on our model, we only hypothesize about causal directions. Mendelian randomization might enable inference of causal direction using SNPs as instrumental variables. Much larger sample sizes are needed than available for this study, though. Ideally, potentially causal edges in our model should be further investigated in dedicated functional studies or randomized clinical trials to establish causality and infer causal direction.

## Conclusion

This is, to our knowledge, the first study integrating data from four omics technologies and clinical phenotypes using an integrated statistical approach. Despite the relatively small sample size, our model confirms causal mechanisms of disease, which have been previously found using highly specific experiments and clinical trials, purely based on observational data from a generally healthy cohort. Moreover, we uncovered several new potential mechanisms that might contribute to disease comorbidities. We found, for instance, urate as key factor connecting body composition and renal function, as well as several phenotypes of the metabolic syndrome. Moreover, by integrating multiple omics datasets, we find the hormone oxytocin as a central mediator that connects inflammation and obesity and, thus, supports the theory of inflammaging.

Our study highlights the importance and the feasibility of data integration across omics layers including phenomics while considering multivariate dependencies. In the future this will help to focus on few, interesting associations, which can then be specifically tested in model organisms and clinical trials. Eventually this will speed up drug discovery by excluding irrelevant pathways and potential drug targets early in the development and thus limiting the set of potential targets and reducing costs of drug discovery.

## Materials and Methods

### Study Population

We analyzed data from the TwinsUK cohort, a national register of 11,000 adult twins recruited as volunteers without selecting for any particular disease or trait. For this study we selected 510 female participants (62 monozygotic twin pairs, 116 dizygotic twin pairs and 154 singletons) aged between 34 and 84 (mean 59.0 ± 9.4) with measurements for epigenomics, transcriptomics, glycomics and metabolomics available. The study has been approved by the local St. Thomas’ Hospital Research Ethics Committee and was carried out in accordance with the approved guidelines. All study participants provided written informed consent.

### Data Acquisition and Processing

The phenotypic data was collected using questionnaires and anthropometric measures during hospital visits. Additionally, four different high-throughput omics datasets were analyzed. With several hundred measured metabolites, thousands of RNA transcripts and particularly hundreds of thousands of CpG sites, network inference is not feasible. We used a knowledge-driven approach to reduce the number of variables from each dataset. To this end, we selected only variables which were previously reported to be strongly (and independently) associated with chronological age as described in the following (and listed in Supplementary Table S1).

#### Epigenomics

DNA methylation levels were measured in adipose tissue samples using Infinium HumanMethylation450 BeadChip (Illumina Inc., San Diego, CA) as previously described^53^. Data was corrected for technical variation using the beta mixture quantile dilation (BMIQ) method and corrected for batch effects and bisulfite conversion levels using linear mixed effect models. Weidner and colleagues^54^ showed that only three aging related differentially methylated regions (aDMRs) are enough to predict the chronological age with high precision. Those three sites, namely cg02228185 (in ASPA), cg25809905 (in ITGA2B) and cg17861230 (PDE4C), were selected for further analyses.

#### Transcriptomics

RNA abundance was measured in abdominal fat samples using the Illumina Human HT-12 V3 Bead chip as part of the MuTHER project as previously described^55^. The probe intensities were adjusted for batch effects using linear models prior to analysis. A previous study found 188 genes (199 probes) significantly associated with chronological^56^ age. We performed stepwise regression to select expression probes independently associated with age. This procedure left 24 probes from 24 different genes (see Supplementary Table S1 for full list) for further analysis.

#### Glycomics

For this study IgG glycans were measured in a high-throughput manner as described by Pucic and colleagues^57^. Briefly, IgG was first isolated from 90μl plasma, the attached glycans were released, labelled with 2-aminobenzamide and analyzed by UPLC. The according chromatograms were divided in 24 glycan peaks (GP), corresponding to 24 glycan structures. The data has been described in detail before^58^. Glycan peaks were global normalized, log transformed and corrected for batch effects using ComBat. It has been shown that a linear combination of only three IgG glycan structures - GP6, GP14 and GP15 - explains 58% percent of the variance in age^8^ and furthermore correlates with several aging associated phenotypes. These three structures were selected for our network analysis.

#### Metabolomics

An untargeted LC/MS and GC/MS platform was used to measure metabolite spectra from plasma and serum samples, respectively. Metabolites were subsequently identified by Metabolon Inc., Durham, USA, using their proprietary database^9^. Metabolite levels were scaled by the run-day median, imputed using the run-day minimum, inverse normalized and corrected for batch effects using linear mixed models with the batch as random intercept. About the half of all known circulating blood metabolites were reported to be associated with chronological age in several large population studies^9^. We selected 22 of these metabolites, which were shown to be independently associated with age and together explain 59% of the variance of chronological age^9^.

#### Clinical Phenotypes

A total of 92 phenotypes was combined with the previously described omics data (listed in Supplementary Table S2). Besides the chronological age, we included 13 body composition variables, measured by *dual-energy X-ray absorptiometry* (DXA), as previously described^59^. In addition to DXA measurements we included common body composition measures, such as height, weight, waist and hip circumferences and body mass index (BMI). Lung function was assessed by measuring the *forced expiratory volume in one second* (FEV1) and the *forced vital capacity* (FVC) using standard spirometry^60^. Biochemical measures of *gamma-glutamyltranserase* (GGT) and *alanine aminotransaminase* (ALAT) were used to determine liver function. We furthermore used the CKD-EPI equation^61^ to *estimate the glomerular filtration rate* (eGFR) from serum creatinine as measure of renal function. Moreover, we included data from various questionnaires, assessing disease states, such as arthritis, asthma and chronic pain. Additionally, questionnaires were used to collect lifestyle parameters. Amongst others, we included data about physical activity and nutrition. Food intake data was collected using an established food frequency questionnaire^62^. Item frequencies were merged into 54 food groups and transformed into orthogonal patterns using principal component analysis^63^. We used the first five principal components, which correspond to five different dietary patterns (Supplementary Table S3), in our model. A complete list of phenotypes is shown in Supplementary Table S2.

#### Data Pre-Processing

We excluded samples with more than 20% missing values and subsequently excluded variables with more than 20% missing values. Remaining missing values were imputed using the mice package^64^. All continuous variables were inverse normalized and categorical variables were dichotomized. To account for family relatedness, we included one variable indicating a unique identifier per family during network inference and removed the according node from the network prior to analysis.

#### Data Availability

The transcriptomics and epigenomics data are available at ArrayExpress (accession number E-TABM-1140 and E-MTAB-1866, respectively). All other TwinsUK omics data are publicly available upon request on the departmental website (http://www.twinsuk.ac.uk/data-access/accessmanagement/).

#### Network Inference

The mixed graphical model was inferred using the Graphical Random Forest (GRaFo) method^21^ with the complementary pairs stability selection (CPSS) modification^65^. Briefly, for each variable all remaining variables were ranked according to their conditional dependence assessed by the random forest variable importance. Consequently, two ranks were calculated for each pair of variables *x* and *y*: one based on the variable importance of *x* for the prediction of *y* and the other based on the importance of *y* for the prediction of *x*. The maximum (i.e. worse) of these two ranks was used as rank of the pair and the best ranking pairs were added as edges of the graphical model. This procedure was repeated for 100 random subsets of the data, each containing the half of all samples, and their complementary set containing the other half of the samples. The resulting 200 graphical models were combined using CPSS^65^ to control the family-wise error rate (FWER). Edges which were contained in more than 80% of all complementary pairs were included in the final model, thereby ensuring FWER <0.05. As effect estimators from random forest, and partial effects in mixed models in general, are non-linear and depend on other variables in the model, there is no estimator for the sign of an edge in our model. We, thus, inferred the signs from regression models, regressing each variable against all others, for visualization purposes.

#### Network Analysis

We analyzed the graphical model as undirected, unweighted network *G = (V, E)*, consisting of a set of vertices *V* and a set of edges *E*.

Several measures were calculated to assess the centrality of nodes in the network. The degree of a node *v* is defined as the number of edges that contain this node, thus assessing its direct associations. The clustering coefficient is the proportion of edges within the neighborhood of *v* that are present in the network. It measures the centrality of *v* within its local neighborhood. In contrast, the betweenness centrality considers indirect associations of *v* and assesses its importance for the network integrity. It is defined as the proportion of all shortest paths that contain *v*^12^. Real-world networks often consist of densely connected modules, so-called clusters or communities that represent functional units within the network^66^. We used an unbiased way to identify clusters within our model, independently from the type of a variable. To this end, we used the algorithm of Brandes and colleagues^67^. It optimizes the modularity score that increases with the number of intra-cluster edges and decreases with the number of inter-cluster edges. Despite the high local clustering, many biological networks are characterized by short average path lengths between nodes. These networks are referred to as small world networks. This concept was formalized by Humphries^22^, who introduced the small word index for networks, that assesses the small-world-ness of a network by comparing its clustering coefficient and average shortest path lengths with an Erdös-Rényi random graph.

#### Network Stability

To test the robustness of our model we investigated the dependence of the network topology on the inference process.

Firstly, we assessed robustness of node centrality as well as module assignments when varying the cutoff for edge inclusion. To this end, we defined different models by including edges that are contained in 20%, 40%, 60%, 80% and 100% of the subsamples, respectively, where 80% corresponds to the original model. Additionally, we analyzed a weighted network^68^ including all edges that were observed in at least one subsample. As a measure of stability of node centrality, we determined the correlation of node degrees and clustering coefficients between the original model and the model in the networks for different edge cutoffs (Supplementary Fig. S4). To assess the stability of module assignments, we calculated the adjusted RAND index^69^ as a measure of similarity between the seven network modules of the original age-mgm with modules identified from the networks that were inferred based on different edge cutoffs. The RAND index assesses the similarity of module assignments by counting the agreements between two different module assignments and adjusting it for the number of agreements that are expected by chance. An adjusted RAND index of 1.0 indicates identity between the module assignments of two networks while values around 0.0 indicate dissimilarity of the assigned modules. In addition, we compared the adjusted RAND indices of the networks for the different edge cutoffs with the background distribution of 1000 randomly sampled module assignments (Supplementary Fig. S4C).

Secondly, we investigated the stability of the network and, in particular, the module assignments depending on the pre-selection of omics variables prior to the model inference. To assess the influence of this selection step on our results we inferred a second model from the same dataset but this time including all metabolomics variables with known chemical identity, thus, completely dispensing variable selection for the metabolomics data. The resulting graph consist of 341 nodes (145 of them from the original model and 196 newly added) connected by 1152 edges. 707 of these edges are amongst the new metabolites, 174 connect one new metabolite with one original variable and 271 edges are amongst original variables, of which 253 are also in the original model. The 63 edges that are missing in the large network compared to the original model are, on average, contained in 58% of the subsamples of the large network, suggesting that they were excluded due to the limited power. We find the added metabolites predominantly peripheral to the age-mgm, with 160 of the 174 edges connecting new metabolites with original variables being amongst metabolites and the remaining 14 with either blood lipid measures or renal function. A graphml file of the large network can be found in the Supplementary Dataset S1. To compare module assignments for the large network with the assignments for the original age-mgm, we restricted the large network to the nodes of the age-mgm. Edges in this network represent conditional dependence, given all other variable in the age-mgm and additionally given the 196 added metabolites. We assigned modules using the spinglass algorithm implemented in the igraph package (as calculating the optimal modularity is computationally expensive for large networks). Module assignments are compared using adjusted RAND index and comparison of detailed module membership (Supplementary Table S4).

The stability of the model depending on the underlying sample sets was assessed by comparing our initial model with models inferred from two disjoint datasets containing either the first or the second twin of each family, respectively. Singletons were distributed randomly across both datasets. The resulting models are provided in graphml format in Supplementary Dataset S1.

All data was analyzed using R (version 3.1.2) along with the randomForest (version 4.6), igraph^70^ (version 1.0.1) and ggplot2^71^ packages. The final network model is available as graphml file in the Supplementary Dataset S1.

## Additional Information

**How to cite this article**: Zierer, J. *et al.* Exploring the molecular basis of age-related disease comorbidities using a multi-omics graphical model. *Sci. Rep.* **6**, 37646; doi: 10.1038/srep37646 (2016).

**Publisher's note:** Springer Nature remains neutral with regard to jurisdictional claims in published maps and institutional affiliations.

## Supplementary Material

Supplementary Information

Supplementary Dataset 1
