# Supplementary material for: Exploring the molecular basis of age-related disease comorbidities using a multi-omics graphical model
Source: Sci Rep. 2016 Nov 25;6:37646. doi: 10.1038/srep37646 (PMC5122881; doi:10.1038/srep37646)
Supplement: Supplementary Information [file srep37646-s1.pdf]

Exploring the molecular basis of age-related disease comorbidities using a multi-omics graphical model

Jonas Zierer[1, 2], Tess Pallister[1], Pei-Chien Tsai[1], Jan Krumsiek[3, 4], Jordana Bell[1], Gordan Lauc[1, 5], Tim D Spector[1], Cristina Menni[1], Gabi Kastenmüller[1, 2, 4]

Supplementary Figure S1. Flowchart illustrating the process of variable selection and network inference.

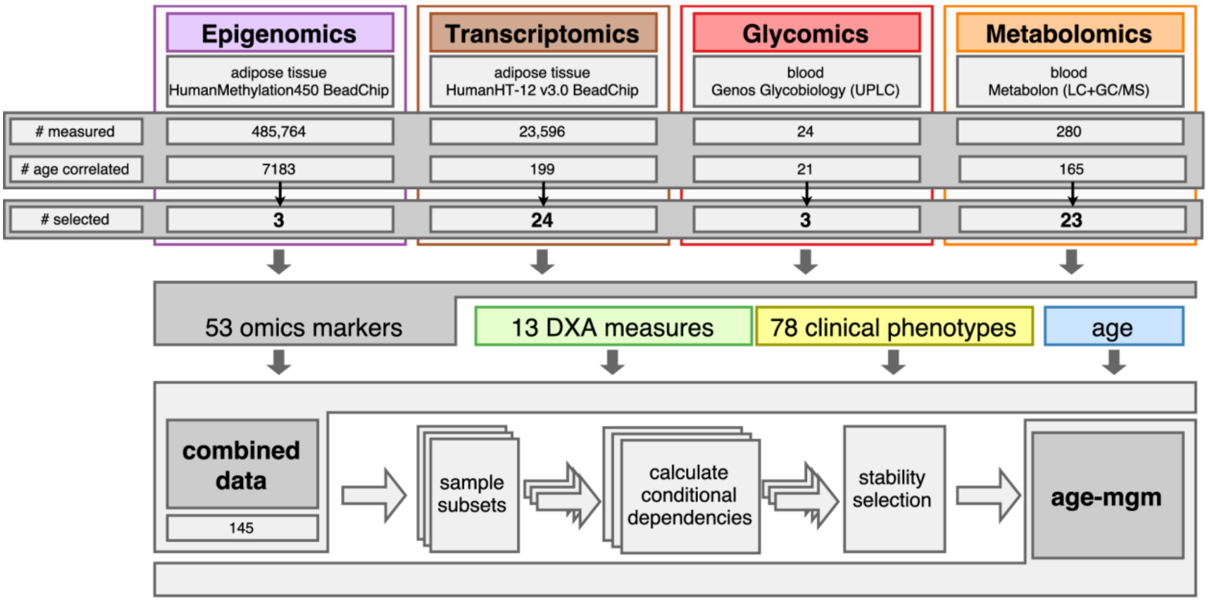

Supplementary Figure S2. Pairwise associations in the age-mgm.

Pairwise associations between variables were assessed using linear and logistic regression models, respectively. Edges in this graph represent associations with FDR < 0.05, edge colors represent the direction of the beta (blue for negative betas, red for positive). Nodes were positioned and colored accordingly to the age-mgm (Fig. 1).

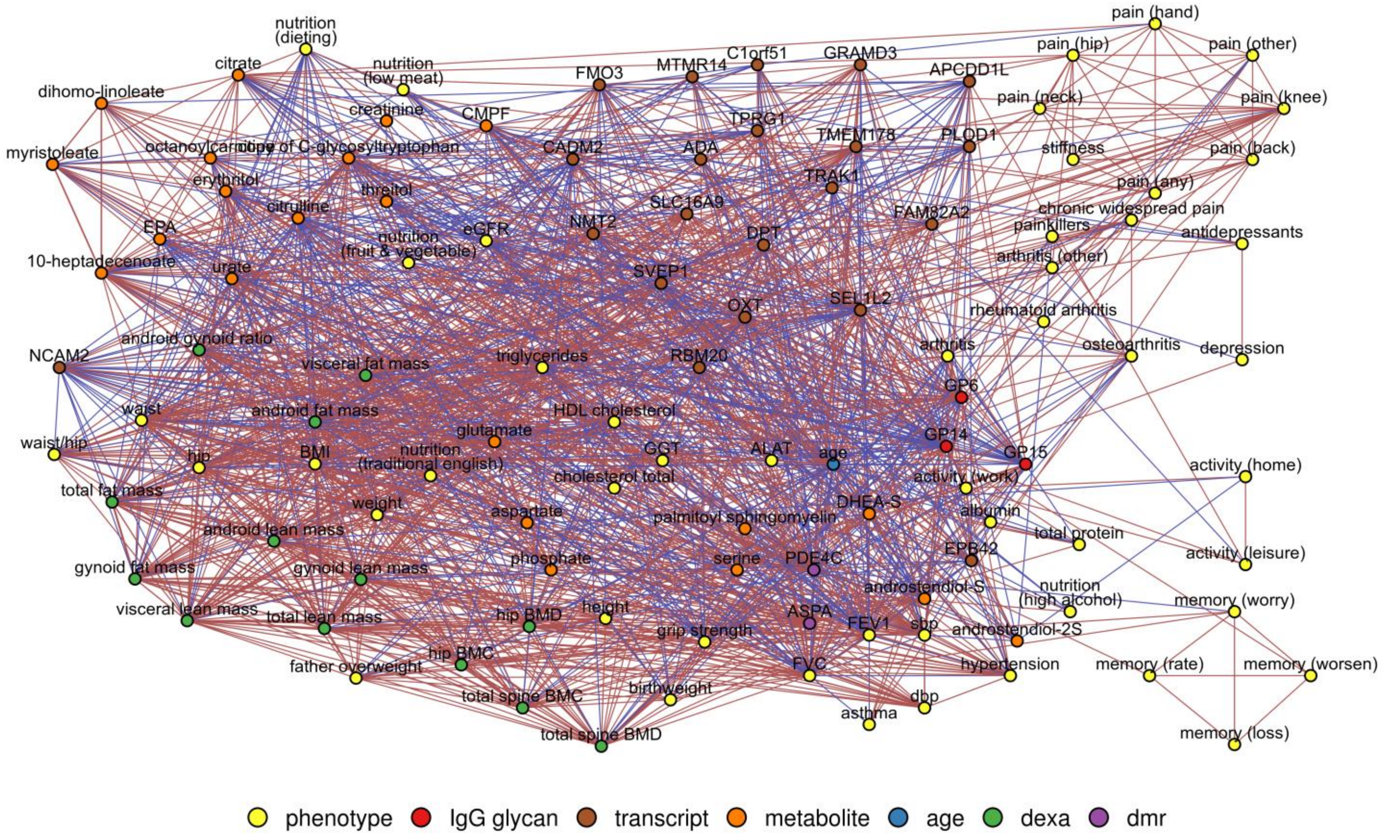

Supplementary Figure S3. Connections within and between groups of variables.

Variables from the age-mgm (Fig. 1) were grouped according to the dataset from which they originate. Node labels indicate the number of variables in each group, while edge labels indicate the total number of edges between groups.

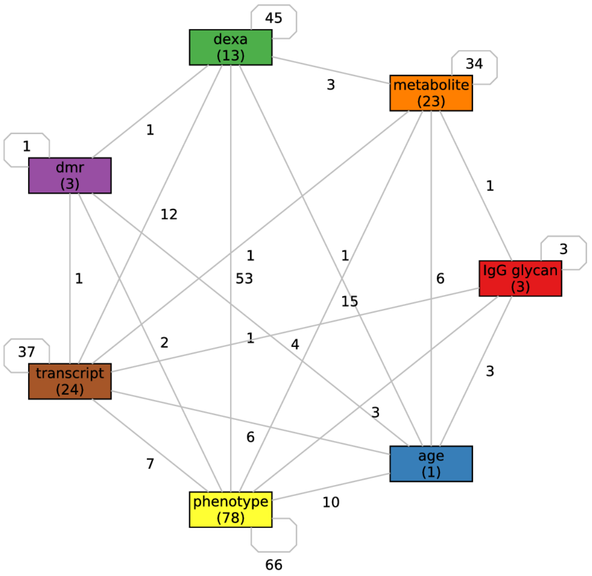

Supplementary Figure S4. Stability of age-mgm regarding edge cutoff.

To assess the dependence of our results on the chosen edge cutoff, we compared measures of node centrality and cluster assignments for networks inferred with varying edge inclusion cutoffs (0.2; 0.4; 0.6; 0.8 (= original cutoff); 1.0) as well as weighted network measures. **(A)** Node degrees and **(B)** clustering coefficients correlate well between the different networks despite their very different sizes (e.g. 969 edges in network with cutoff 0.2 (= very loose cutoff) compared to 185 edges when using cutoff 1.0 (= very stringent cutoff)). **(C)** The adjusted RAND index, which assesses the similarity of module assignments (with a RAND index of 1.0 indicating identity between module assignments and low values indicating dissimilarity between assignments), is above 0.4 for all edge inclusion cutoffs. Thus, comparing the adjusted RAND indices of the networks for the different edge cutoffs with the background distribution of 1000 randomly sampled module assignments yielding indices around 0.0 illustrates the stability of our network inference approach against various choices for edge cutoffs.

(A) Node degrees

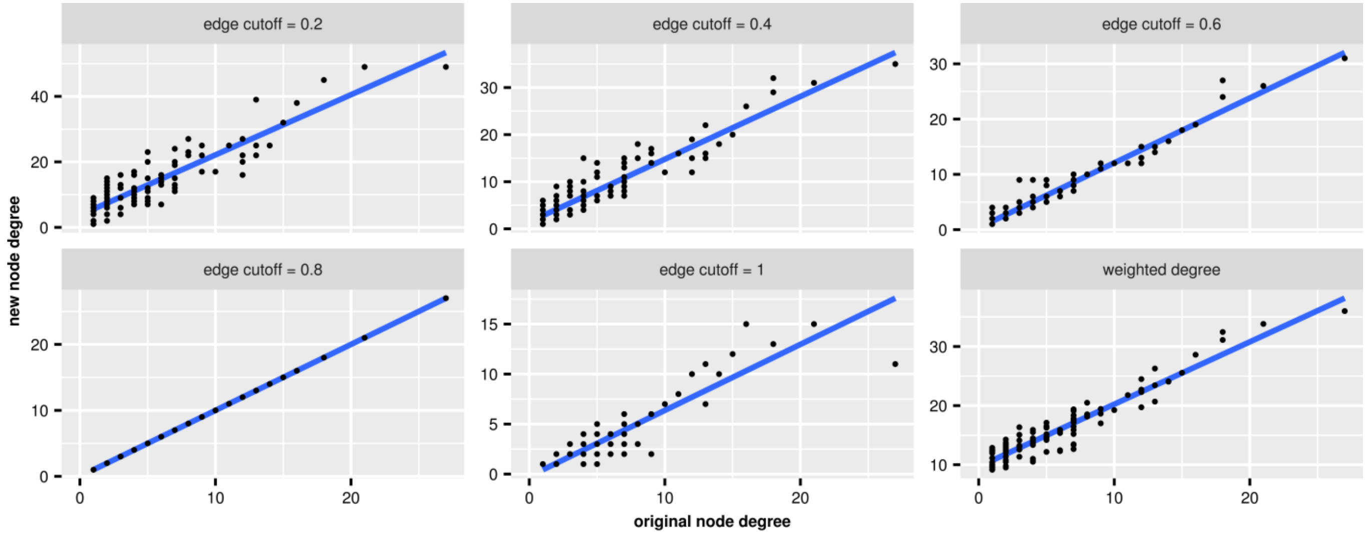

(B) Clustering coefficients

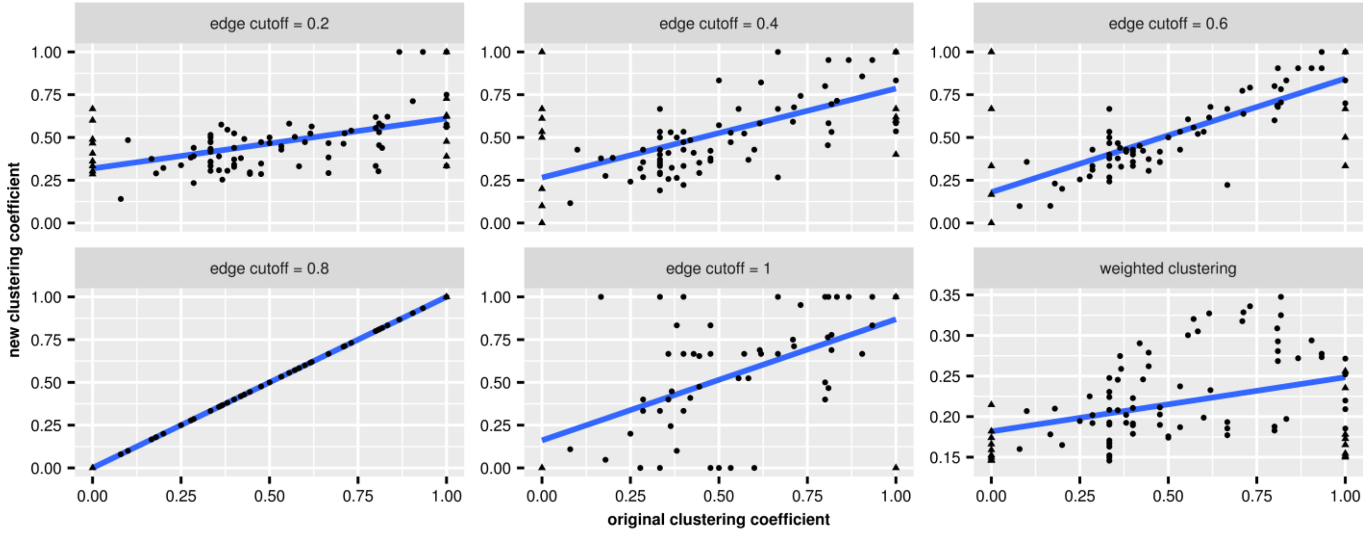

(C) Module Assignments

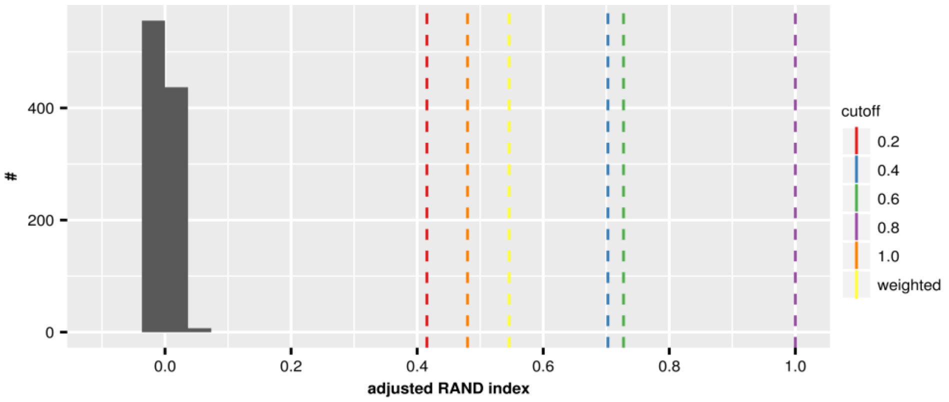

Supplementary Figure S5. Stability of age-mgm across datasets.

To assess the stability of the age-mgm, we additionally inferred two separate models, *twin 1 only* and *twin 2 only*, from two disjoint subsets of our data, each of them containing one member of each twin pair. **(A)** Illustration of the age-mgm showing only edges that are exclusive to the model based on the combined dataset (green), the *twin 1 only* (orange) or the *twin 2 only* (purple) models, respectively. With only 21 edges being unique to the original model the network can be considered stable between the datasets. **(B)** Distribution of the 21 unique edges in the original model across the portion of graphs that contained the edge in the network inference procedure for *twin 1 only* (orange) and *twin 2 only* (purple) models. The edges that are unique to the original model are contained in the majority of subsamples from the twin 1 and twin 2 datasets (78% on average) and, thus, just missed the edge inclusion cutoff of 80% in most cases. The *twin 1 only* and *twin 2 only* models are available as graphml files in the Supplementary Dataset 1.

(A)

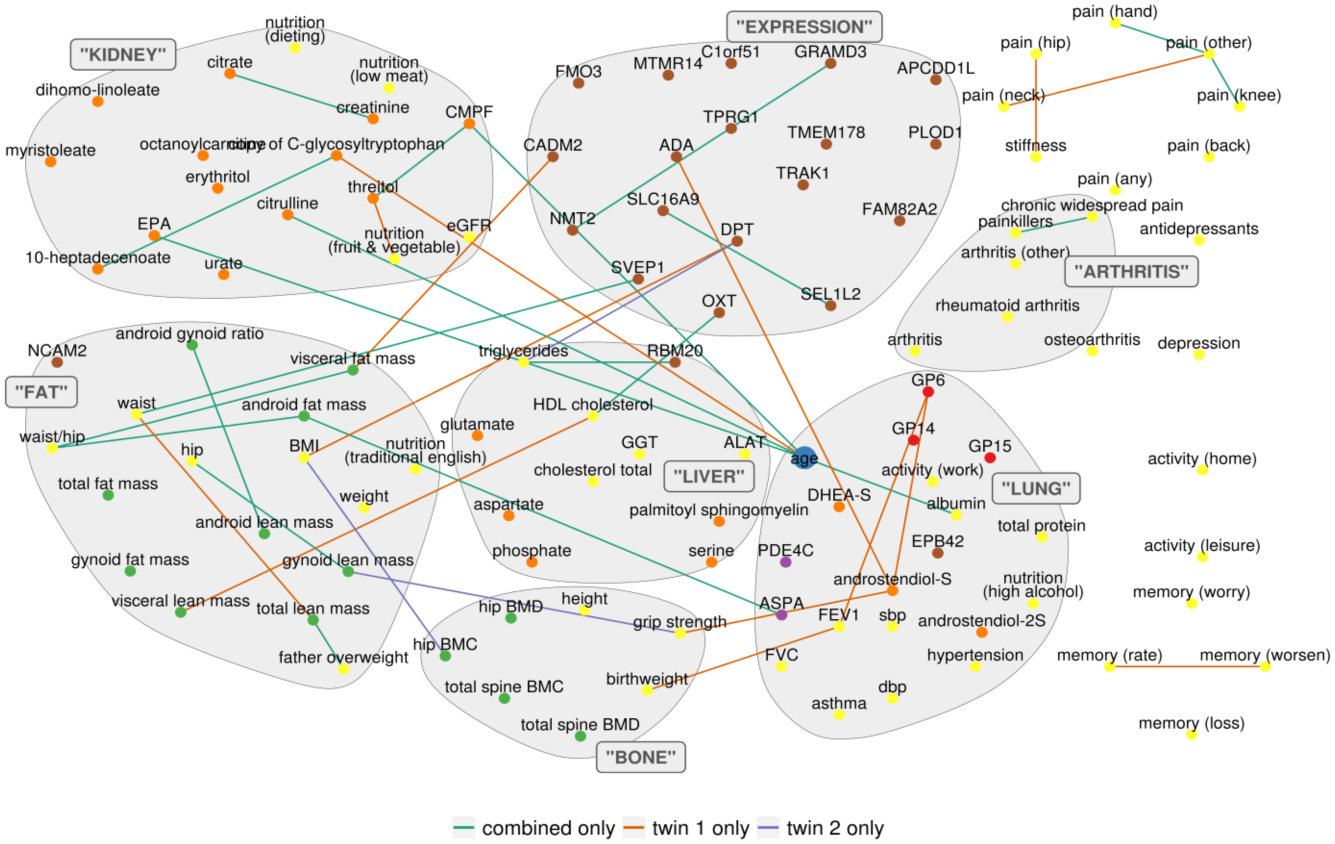

(B)

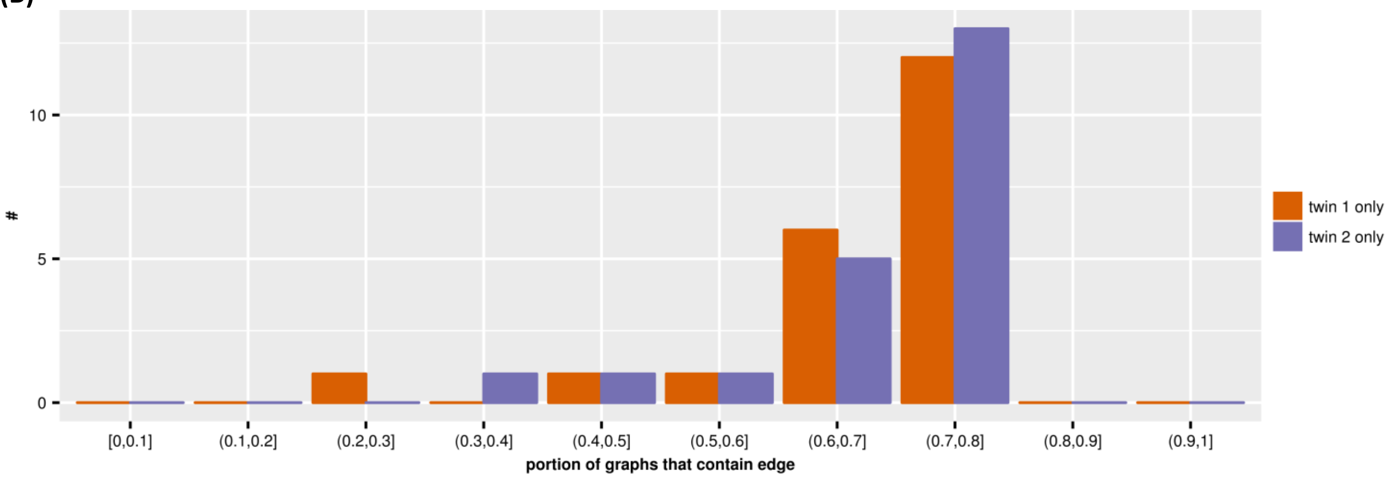

Supplementary Table S1. Full list of omics markers used for the age-mgm.

Degree and clustering coefficients were calculated from the entire model, while the betweenness was calculated for the giant component of the age-mgm only.

Type	Marker	Description	Degree	Clustering Coefficient	Betweenness Centrality
Epigenomics	cg17861230	PDE4C (chr19, 18343901)	5	40.00 %	1.65 %
	cg02228185	ASPA (chr17, 3379567)	2	0.00 %	0.74 %
	cg25809905	ITGA2B (chr17, 42467728)	0		
Transcriptomics	ILMN_2062620	NMT2 (chr10, 15187951:15188000)	13	17.95 %	6.10 %
	ILMN_1735124	OXT (chr20, 3001023:3001072)	9	33.33 %	6.03 %
	ILMN_2112638	SVEP1 (chr9, 112168560:112168609)	9	27.78 %	10.47 %
	ILMN_1750018	SEL1L2 (chr20, 13778194:13778243)	8	28.57 %	4.46 %
	ILMN_1684391	PLOD1 (chr1, 11958106:11958155)	7	38.10 %	0.31 %
	ILMN_1708107	DPT (chr1, 166931502:166931551)	7	33.33 %	2.58 %
	ILMN_2065690	GRAMD3 (chr5, 125857223:125857272)	6	53.33 %	0.24 %
	ILMN_1678403	TMEM178 (chr2, 39797960:39798009)	5	60.00 %	0.47 %
	ILMN_1790350	TPRG1 (chr3, 190523591:190523640)	5	20.00 %	3.30 %
	ILMN_1664679	CADM2 (chr3, 86200275:86200324)	4	33.33 %	0.96 %
	ILMN_1727309	FAM82A2 (chr15, 38815707:38815756)	4	16.67 %	0.22 %
	ILMN_1732410	SLC16A9 (chr10, 61080824:61080873)	4	33.33 %	2.35 %
	ILMN_1782069	TRAK1 (chr3, 42228905:42228954)	4	50.00 %	0.18 %
	ILMN_1803686	ADA (chr20, 42681856:42681905)	4	33.33 %	2.87 %
	ILMN_2344283	FMO3 (chr1, 169353283:169353332)	4	50.00 %	0.34 %
	ILMN_1749962	NCAM2 (chr21, 21832517:21832566)	3	100.00 %	0.00 %
	ILMN_1689431	APCDD1L (chr20, 56467867:56467916)	2	100.00 %	0.00 %
	ILMN_1749540	RBM20 (chr10, 112588928:112588977)	2	0.00 %	1.45 %
	ILMN_1814397	EPB42 (chr15, 41276902:41276951)	2	0.00 %	2.11 %
	ILMN_2297864	MTMR14 (chr3, 9714444:9714493)	2	100.00 %	0.00 %
	ILMN_1793543	C1orf51 (chr1, 148525842:148525891)	1		0.00 %
	ILMN_1657087	ZNF533 (chr2, 180015130:180015179)	0		
	ILMN_1727833	KIF19 (chr17, 69858798:69858847)	0		
	ILMN_1794552	GAP43 (chr3, 116922460:116922509)	0		
Glycomics	GP6	The percentage of FA2B glycan in total IgG glycans	7	38.10 %	1.80 %
	GP14	The percentage of FA2G2 glycan in total IgG glycans	7	47.62 %	1.83 %
	GP15	The percentage of FA2BG2 glycan in total IgG glycans	3	100.00 %	0.00 %
Metabolomics	C-gly-trp	Amino Acid	9	25.00 %	3.93 %
	citrate	Energy	7	47.62 %	0.44 %
	DHEA-S	Lipid	7	28.57 %	4.95 %
	10-heptadecenoate	Lipid	6	66.67 %	0.17 %
	CMPF	Lipid	6	33.33 %	4.23 %
	EPA	Lipid	6	40.00 %	5.77 %
	dihomo-linoleate	Lipid	5	80.00 %	0.00 %
	erythritol	Xenobiotics	5	40.00 %	0.99 %
	glutamate	Amino Acid	5	10.00 %	8.26 %
	myristoleate	Lipid	5	80.00 %	0.98 %
	octanoylcarnitine	Lipid	5	80.00 %	0.00 %
	urate	Nucleotide	5	40.00 %	4.20 %
	threitol	Carbohydrate	4	33.33 %	3.67 %
	androstendiol-2S	Lipid	3	33.33 %	1.16 %
	creatinine	Amino Acid	3	66.67 %	0.29 %
	phosphate	Energy	3	33.33 %	0.09 %
	androstendiol-S	Lipid	3	66.67 %	1.38 %
	citrulline	Amino Acid	2	0.00 %	0.00 %
	palmitoyl sphingomyelin	Lipid	2	0.00 %	0.22 %
	serine	Amino Acid	2	0.00 %	0.20 %
	aspartate	Amino Acid	1		0.00 %
	1,7-dimethylurate	Xenobiotics	0		
	creatine	Amino Acid	0		

Supplementary Table S2. List of all clinical phenotypes used for the age-mgm.

The second column contains the average value and standard deviation within the study population in case of continuous variables and the prevalence of a phenotype in case of binary variables. Degree, clustering and betweenness were calculated from the entire model, where betweenness was calculated from the giant component *age-mgm* only.

Phenotype	value	Degree	Clustering Coefficient	Betweenness Centrality
age, years	59.03+-9.40	27	7.98 %	47.49 %
android fat mass[1]	2284.44+-1022.14	21	36.67 %	9.13 %
BMI, kg/m2	26.63+-4.87	18	44.44 %	10.11 %
visceral fat mass[1]	1242.36+-566.78	18	44.44 %	2.49 %
weight, kg	69.44+-13.67	16	58.33 %	1.76 %
android gynoid ratio [1]	0.96+-0.16	15	41.90 %	3.18 %
gynoid lean mass[1]	6195.86+-934.68	14	57.14 %	1.45 %
total lean mass[1]	9272.55+-1436.57	14	61.54 %	2.44 %
android lean mass[1]	3076.08+-550.41	13	73.08 %	2.26 %
total fat mass[1]	6997.33+-2298.12	13	80.77 %	0.09 %
gynoid fat mass[1]	4712.17+-1392.56	12	81.82 %	0.00 %
height, cm	161.40+-5.82	12	36.36 %	4.96 %
hip, cm	101.53+-9.77	12	71.21 %	3.95 %
visceral lean mass[1]	1785.70+-288.29	12	81.82 %	0.08 %
waist, cm	80.08+-10.40	11	70.91 %	1.36 %
hip BMC[1]	32.34+-8.60	10	55.56 %	0.74 %
FEV1, l	2.53+-0.56	8	35.71 %	4.34 %
triglycerides, mmol/L	0.01+-0.20	8	35.71 %	3.74 %
FVC, l	3.20+-0.63	7	47.62 %	2.20 %
eGFR, mL/min/1.73m2	81.84+-14.99	7	28.57 %	2.14 %
hip BMD[1]	0.92+-0.20	7	42.86 %	7.36 %
pain (any)	19.60% (89/454)	7	80.95 %	
pain (back)	11.23% (51/454)	7	80.95 %	
pain (knee)	11.89% (54/454)	7	80.95 %	
total spine BMC[1]	59.01+-12.71	7	61.90 %	0.08 %
waist/hip[1]	0.79+-0.05	7	90.48 %	0.00 %
grip strength, kg	26.89+-6.20	6	53.33 %	1.75 %
pain (hand)	10.35% (47/454)	6	93.33 %	
pain (hip)	9.47% (43/454)	6	93.33 %	
pain (neck)	7.05% (32/454)	6	86.67 %	
HDL cholesterol, mmol/L	1.81+-0.47	5	40.00 %	2.36 %
pain (other)	4.85% (22/454)	5	100.00 %	
SBP, mm Hg	131.16+-17.71	4	33.33 %	4.17 %
arthritis	39.27% (183/466)	4	33.33 %	2.11 %
cholesterol total, mmol/L	5.61+-1.09	4	33.33 %	1.19 %
osteoarthritis	23.96% (115/480)	4	33.33 %	6.18 %
stiffness	5.29% (24/454)	4	100.00 %	
total spine BMD[1]	0.99+-0.16	4	83.33 %	0.00 %
memory (worry)	34.27% (170/496)	3	33.33 %	
nutrition (fruit & vegetable) [1]	0.74+-2.06	3	100.00 %	0.00 %
DBP, mm Hg	79.30+-9.85	2	100.00 %	0.00 %
GGT, U/L	25.70+-21.58	2	0.00 %	2.11 %
albumin, g/L	44.20+-3.64	2	0.00 %	2.11 %
arthritis (other)	13.04% (57/437)	2	100.00 %	0.00 %
chronic widespread pain	24.88% (102/410)	2	0.00 %	2.11 %
father overweight	10.79% (49/454)	2	100.00 %	0.00 %
hypertension	23.53% (120/510)	2	100.00 %	0.00 %
memory (loss)	36.29% (168/463)	2	100.00 %	
memory (rate)	65.66% (325/495)	2	100.00 %	
nutrition (low meat) [1]	-0.19+-1.25	2	0.00 %	2.11 %

Phenotype	value	Degree	Clustering Coefficient	Betweenness Centrality
activity (home)	71.12% (330/464)	1		
activity (leisure)	63.15% (293/464)	1		
activity (work)	63.79% (296/464)	1		0.00 %
antidepressants	17.32% (71/410)	1		
asthma	13.53% (69/510)	1		0.00 %
birthweight, g	2381.64+-545.03	1		0.00 %
depression	10.80% (50/463)	1		
memory (worsen)	27.22% (135/496)	1		
nutrition (dieting)[1]	0.28+-1.28	1		0.00 %
nutrition (high alcohol) [1]	0.07+-1.39	1		0.00 %
nutrition (traditional English) [1]	0.26+-1.49	1		0.00 %
painkillers	12.93% (53/410)	1		0.00 %
rheumatoid arthritis	4.90% (25/510)	1		0.00 %
total protein, g/L	70.02+-4.44	1		0.00 %
ankle swelling	5.29% (24/454)	0		
anxiety stress disorder	14.69% (68/463)	0		
bilirubin, mmol/L	8.96+-4.40	0		
cancer	12.16% (62/510)	0		
chest pain	19.41% (99/510)	0		
chronic bronchitis	7.03% (31/441)	0		
dizziness	4.71% (24/510)	0		
glucose, mmol/L	4.94+-0.52	0		
hearing	37.98% (177/466)	0		
heart murmur	5.40% (25/463)	0		
high cholesterol	27.06% (138/510)	0		
irregular heartbeats	5.40% (25/463)	0		
learn gadgets	28.63% (142/496)	0		
loss of vision/speech	6.70% (31/463)	0		
low back pain	11.46% (47/410)	0		
memory (clothes)	6.05% (30/496)	0		
memory (keys)	38.51% (191/496)	0		
memory (medication)	10.89% (54/496)	0		
memory (month)	11.90% (59/496)	0		
migraine	17.06% (79/463)	0		
mother overweight	17.40% (79/454)	0		
nutrition (total energy), kcal	1893.71+-575.12	0		
palpitations	8.15% (37/454)	0		
smoking	10.20% (52/510)	0		
telomere length[1]	3.59+-0.63	0		
thyroid underactive	7.84% (40/510)	0		
varicose veins	19.22% (98/510)	0		

[1] these measurements do not have a unit

BMI = body mass index, BMC = bone mineral content, BMD = bone mineral density, FEV = forced expiratory volume, FVC = forces vital capacity, eGFR = estimated glomerular filtration rate, HDL = high-density lipoprotein, SBP = systolic blood pressure, DBP = diastolic blood pressure, GGT = gamma-glutamyl transpeptidase, ALAT = alanine aminotransferase

Supplementary Table S3. Principle component-derived dietary patterns, with percentage of variance explained, and foods consumed in high and low intakes.

Diet pattern	Variance explained	High intakes[1]	Low intakes[2]
Fruit & vegetable	8.20%	Fruit, allium and cruciferous vegetables	Fried potatoes
High alcohol	3.90%	Beer, wine and allium vegetables	High fiber breakfast cereals and fruit
Traditional English	3.60%	Fried fish and potatoes, meats, savoury pies and cruciferous vegetables	
Dieting	3.30%	Low-fat dairy products, low-sugar soda	Butter and sweet baked products
Low meat	3.20%	Baked beans, pizza and soy foods	Meat, other fish and seafood, and poultry

[1] Food frequency questionnaire items with factor loadings ≥0.20.
[2] Food frequency questionnaire items with factor loadings ≤-0.20.

Supplementary Table S4. Stability of age-mgm against variable selection procedure.

To assess the influence variable selection prior to network inference on our results we inferred a second model from the same dataset but without pre-selecting metabolomics variables. We then restricted the large network to the nodes of the *age-mgm*. Edges in this network represent conditional dependence, given all other variables in the age-mgm and additionally given the 196 added metabolites. While the underlying larger model is very different from the original one in terms of size, sparsity and included variables, we find the network modules stable across both networks (adjusted RAND index 0.57). Detailed module memberships are listed for this network and the original age-mgm. Variables in italics have degree 0 in the large network and consequently not part of any cluster

Module	Members in *age-mgm*	Members in *large-mgm*
**EXPRESSION**	CADM2, TMEM178, PLOD1, APCDD1L, DPT, FAM82A2, SLC16A9, OXT, SEL1L2, TRAK1, TPRG1, C1orf51, ADA, NMT2, GRAMD3, SVEP1, MTMR14, FMO3	CADM2, TMEM178, PLOD1, APCDD1L, FAM82A2, TRAK1, ADA, NMT2, GRAMD3, SVEP1, MTMR14, FMO3<br><br>SLC16A9, TPRG1, C1orf51
**LUNG**	FEV1, FVC, GP14, GP15, GP6, EPB42, androstendiol-S, DHEA-S, androstendiol-2S, activity (work), age, *albumin*, *asthma*, ASPA, PDE4C, DBP, hypertension, *nutrition (high alcohol)*, SBP, *total protein*	FEV1, FVC, GP14, GP15, GP6, OXT, RBM20, SEL1L2, EPB42, androstendiol-S, DHEA-S, activity (work), age, ASPA, PDE4C, DBP, hypertension, SBP
**ARTHRITIS**	arthritis, arthritis (other), chronic widespread pain, osteoarthritis, *painkillers*, rheumatoid arthritis	arthritis, arthritis (other), chronic widespread pain, osteoarthritis, rheumatoid arthritis
**BONE**	birthweight, grip strength, height, hip BMC, hip BMD, total spine BMC, total spine BMD	birthweight, grip strength, gynoid lean mass, height, hip BMC, hip BMD, total spine BMC, total spine BMD
**FAT**	NCAM2, android fat mass, android gynoid ratio, android lean mass, BMI, *father overweight*, gynoid fat mass, gynoid lean mass, hip, *nutrition (traditional english)*, total fat mass, total lean mass, visceral fat mass, visceral lean mass, waist, waist/hip, weight	DPT, NCAM2, android fat mass, android gynoid ratio, android lean mass, BMI, gynoid fat mass, hip, total fat mass, total lean mass, visceral fat mass, visceral lean mass, waist, waist/hip, weight
**LIVER**	ALAT, GGT, RBM20, phosphate, aspartate, serine, glutamate, palmitoyl sphingomyelin, HDL cholesterol, cholesterol total, triglycerides	ALAT, GGT, phosphate, aspartate, serine, glutamate, androstendiol-2S, palmitoyl sphingomyelin, HDL cholesterol, cholesterol total, triglycerides
**KIDNEY**	creatinine, *citrate*, urate, *citrulline*, *dihomo-linoleate*, *EPA*, erythritol, *CMPF*, *myristoleate*, C-gly-trp, *octanoylcarnitine*, *10-heptadecenoate*, threitol, eGFR, *nutrition (fruit & vegetable)*, *nutrition (dieting)*, *nutrition (low meat)*	creatinine, urate, erythritol, C-gly-trp, threitol, eGFR
